# Performance Evaluation of Resource Management in Cloud Computing Environments

**DOI:** 10.1371/journal.pone.0141914

**Published:** 2015-11-10

**Authors:** Bruno Guazzelli Batista, Julio Cezar Estrella, Carlos Henrique Gomes Ferreira, Dionisio Machado Leite Filho, Luis Hideo Vasconcelos Nakamura, Stephan Reiff-Marganiec, Marcos José Santana, Regina Helena Carlucci Santana

**Affiliations:** 1 ICMC, University of São Paulo, São Carlos, SP, Brazil; 2 Department of Computer Science, University of Leicester, Leicester, United Kingdom; Nankai University, CHINA

## Abstract

Cloud computing is a computational model in which resource providers can offer on-demand services to clients in a transparent way. However, to be able to guarantee quality of service without limiting the number of accepted requests, providers must be able to dynamically manage the available resources so that they can be optimized. This dynamic resource management is not a trivial task, since it involves meeting several challenges related to workload modeling, virtualization, performance modeling, deployment and monitoring of applications on virtualized resources. This paper carries out a performance evaluation of a module for resource management in a cloud environment that includes handling available resources during execution time and ensuring the quality of service defined in the service level agreement. An analysis was conducted of different resource configurations to define which dimension of resource scaling has a real influence on client requests. The results were used to model and implement a simulated cloud system, in which the allocated resource can be changed on-the-fly, with a corresponding change in price. In this way, the proposed module seeks to satisfy both the client by ensuring quality of service, and the provider by ensuring the best use of resources at a fair price.

## Introduction

In recent years, cloud computing has been one of the most widely discussed topics in IT (Information Technology). According to NIST (National Institute of Standards and Technology), “*Cloud computing is a model that allows ubiquity, convenience and on-demand access to a shared pool of configurable resources and can be quickly delivered with minimum managerial effort on the part of the clients*” [1].

Cloud computing is a computing model in which resource providers can offer on-demand services for clients in a transparent way, on a pay-as-you-use basis. It introduces a new level of flexibility and scalability in IT organizations and allows clients and providers to deal with rapid changes in IT scenarios where there is a need to reduce costs and time by employing an infrastructure management solution. However, as the cloud is a distributed system that provides services, the computational system that covers this environment is expected to operate properly, with no interruption in its service, or data loss. This means that service providers should ensure Quality of Service (QoS) to win the confidence of their clients and give them the expected satisfaction.

The term quality of service refers to the service performance, composed of performance of individual features, that determines the degree of client satisfaction, i.e., the features of the system that are required to meet the client’s requirements [2]. These characteristics are attributes of a system that can be measured quantitatively by metrics and used to define QoS levels [3]. However, ensuring QoS in a cloud environment is not a trivial task, since there are different types of clients with varied service requirements, that operate in an environment that is based on the Internet. Furthermore, different providers can offer the same service by deploying different technologies.

As discussed in [4], resource matching and issues about making recommendations have often been neglected, such as the use of attribute weights and the collaborative application of empirical data, marginal utility, and QoS constraints. The collection and combination of data in assessing the reliability of the cloud service is also challenging, since QoS values may be missing in an offline situation, because they are time-consuming and the cloud service invocation is expensive, as Ding et al. [5] and Ding et al. [6] make clear.

With regard to the question of scalability in a cloud environment, where the demand for services can change at almost any time, automatic resource allocation to meet this demand has become a key issue both in the academic world and industry. Correct provisioning allows a better use of available computational resources and, hence, of the whole infrastructure that comprises the cloud, because the system mapping between the workload and resources is more efficient. Furthermore, it helps to achieve the QoS levels required by the clients and makes the system more dynamic.

For instance, providing more computational resources to a client is really feasible and easily achieved in a cloud environment, since the virtualized computational resources are regarded by clients as being unlimited. However, the provisioning of more computational resources to meet the requests increases the final cost that is paid by the client and requires the provider to employ efficient mechanisms.

Providers such as Amazon EC2 and Microsoft Azure employ a resource provisioning methodology in which clients are responsible for estimating the amount of resources needed and selecting the instance [7]. The Virtual Machines (VMs) are responsible for executing the clients’ requests and are arranged in classes according to their configurations of memory, virtual cores and disk size. The price is defined as being based on the class, where the most powerful instances are the most expensive.

Owing to the scaling process, it is striking that an increase in the number of resources does not necessarily lead to the best performance. However, this increase affects the costs. For example, if an application needs significantly more CPU power, but has no additional memory, and the next VM class provides an average increase in both areas (CPU and memory), the client can change from a VM type to another one with more resources. In this way, they can obtain a better performance by paying more. However, this increases all resources, while in practice all that might be required is an increase in CPU capacity in order to execute the application with the required QoS—hence unneccesary waste of other resources and costs for the user are imposed by the current model.

The above mentioned providers do not change the VMs configurations on-the-fly. They change the number of instances respecting the classes defined by them, by either adding or removing VMs of the same class or VMs of different classes.

In our view, the resource allocation in a cloud can be performed automatically and dynamically a) by addressing the high-level needs of clients at a fair price, and b) where clients can set up VMs with different configurations, instead of being restricted to the VMs classes defined by providers, they must be able to define the number of virtual cores, memory or disk size, for instance, that they want to contract at a fair price.

This is not an easy undertaking because it requires taking account of some key parameters such as budgeted resources, time constraints, and/or the desired quality of service. Furthermore, there are unpredictable situations that can impair the efficiency of the provisioning services and delivery during the execution time, such as demand estimation with expenditure measurement errors, dynamic workload and unpredictable behavior of the system [8].

The wrong combination of computational resources and applications leads to either an under or over-estimation of client demand and has an effect on the contracted QoS and on the service costs. Moreover, highly variable load spikes can occur on demand, depending on the day and time of year, and the popularity of an application. These factors give rise to problems in workload behavior estimation and related requirements for resources. Finally, the availability, workload, and throughput of resources and network connections can vary in an unpredictable way in a large-scale computing environment such as the cloud. The instability in the above mentioned system makes it hard to determine what resources are required during the provisioning process.

For this reason, this paper outlines a module for resource management in a cloud environment that examines how to handle the available resources on-the-fly and the effect of this manipulation on both the performance of the system and the business model. The proposed module, called ReMM—Resource Management Module, is a dynamic and self-managed module that is responsible for load balancing, efficient utilization of resources and QoS level assurance. It adopts an approach that involves varying the VMs resource capabilities and applies both vertical and horizontal scalability, by changing the number of resources that compose a VM and the number of instances, when necessary.

A performance analysis is conducted, in which the computational resources and the number of these resources that have to be allocated to a client in a cloud with different workloads are defined. In this way, it is possible to quantify the influence of configurations of a different number of VMs and virtual cores (vCPUs), disk size, network type and memory RAM capacity on the performance of the system.

On the basis of influence of the resources, we simulated an environment to validate our proposed module. Following this, we showed the impact on the execution mean time and on the cost to contract a specific VM when there were changes in the configuration of the resources. The results show that our module effectively changes the available resources on-the-fly, by ensuring the QoS contracted with proportional changes to the cost.

To summarize the previous paragraphs, the main novel contributions of this paper are:

- A: a detailed performance analysis leading to conclusions of key influences on executions;
- B: a novel mechanism and management module to enable dynamic scalability in one resource dimension;
- C: an evaluation showing the positive influence of the new module.

The remainder of this paper is structured as follows: the Section on Related Work conducts a brief analysis of the works available in the literature; Section ReMM—Resource Management Module outlines the proposed module that is deployed with the aid of CloudSim simulator [9]; the experiments that show the benefits of using ReMM were carried out and analysed in the Section called Performance Evaluation; finally, there is the Conclusion and Future Work which summarizes the main results and make recommendations for future work, respectively.

## Related Work

The complexity of finding an optimum resource allocation is exponential in huge systems such as big clusters, grids and cloud data centers. Since resource demand and supply can be dynamic and uncertain, various strategies for resource management are available in the literature [8].

In [10], for instance, the authors carry out a study about the allocation of resources among multiple HPC (High Performance Computing) systems such as cluster, grid and cloud. Sharkh et al. [11] discuss various internal and external factors that should be considered in the resources allocation process. Manvi and Shyam [12] conduct a study that examines resource management techniques such as provisioning, allocation, mapping and adaptation in an Infrastructure as a Service. In [13], various resource allocation strategies and their challenges are discussed.

The authors in [14] explore adaptive resource allocation for back-end mashup applications. In the developed prototype, the back-end is a resource-intensive system responsible for the continuous collection and analysis of real-time data from external services or applications. On the basis of this analysis, the prototype adaptively allocates resources to the back-ends to meet the clients’ requests. However, in the shown results, there is only a kind of virtual machine with fixed configurations that is assigned to meet any demands. Thus, there is no heterogeneity in the VM configurations and no runtime adaptation in the VM capabilities. Another limitation of this prototype is the lack of scaling down capability as the number of requests made by active clients declines.

The work shown in [7] proposes a cost-based approach for allocating resources to workflow-based applications which employs four strategies for the allocation of a virtual infrastructure. However, as one virtual machine is deployed per physical machine and all the physical machines have the same configuration, the available resources can be overloaded or idle owing to the heterogeneity of the application. Another limitation is that the prototype is not dynamic, because it is not possible to add or remove instances or even change the VM capabilities during the execution time.

Inomata et al. [15] propose a dynamic and not self-managed architecture, in which the clients are responsible for adding or removing instances by commands in accordance with the workload. However, the prototype has only been set up for one type of instance and there is no runtime adaptation in the VMs capabilities.

In [16], the authors propose resource allocation algorithms for SaaS (Software as a Service) providers that are designed to reduce the costs of the infrastructure and Service Level Agreement (SLA) violations. However, the simulated environment does not change the capabilities of the VMs. It seeks to maximize profits by reusing the created VMs. In view of this, the proposed algorithms attempt to assign new requests to the created VMs, by adopting a multi-tenancy approach, which can violate the SLA.

Calheiros, Ranjan and Buyya [8] propose an adaptive approach to investigate the automated task management and resource scalability to ensure the QoS contracted by clients. The authors put forward a simple load-balancing policy for resource provisioning, in which new VMs are created with fixed configurations in the host where there are fewer running applications. However, there are no changes in the VM capabilities during the request execution time.

In the analysed papers, the authors employ different mechanisms for resource management, but in all of them, there are no changes on-the-fly in the available resources. In this way, they are able to apply a horizontal scalability. The module proposed in this paper takes account of both horizontal and vertical scalability. Thus, in examining the scalability in a cloud environment, where the demand for services changes all the time, an environment with fixed resources may not be the most efficient when the question of the use of resources and client satisfaction are taken into account.

## ReMM—Resource Management Module

The module proposed in this paper, called ReMM, concerns the way the available resources are handled during the execution time with regard to the QoS metrics defined in the SLA. We outline a resource management module for cloud applications in which both horizontal and vertical scalability can be applied dynamically, thus leading to a change in price.

**Fig 1:**
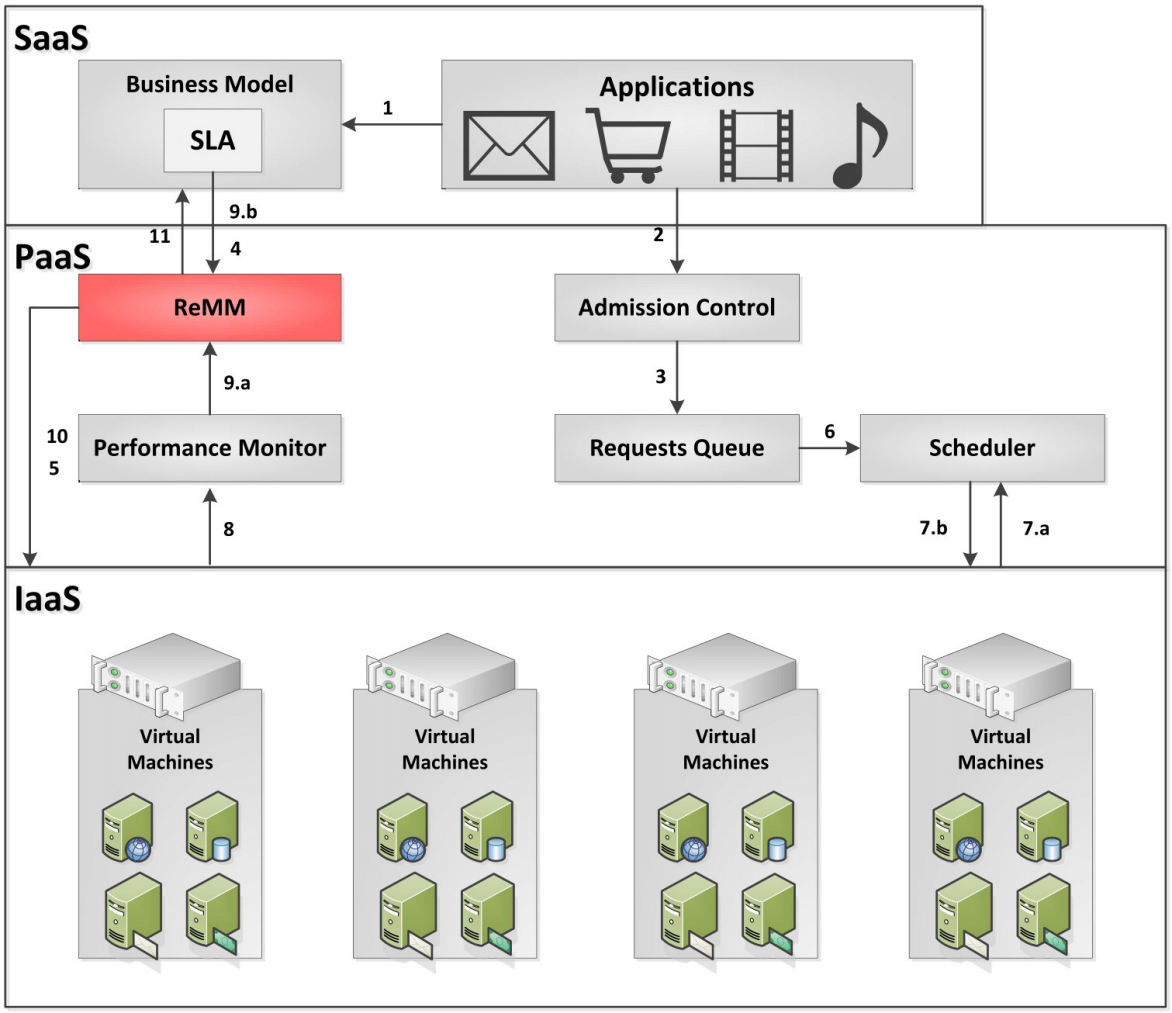
ReMM operation. Depending on the demand for the service and clients’ requests, the ReMM may change the configuration of the resources while at the same time changing the price.

The most common type of scaling is horizontal scaling which involves the allocation and release of virtual machines. The other type is vertical scaling which either increases or reduces the computing resources (vCPU, memory, disk, etc.) of one or more instances [17]. In this way, the ReMM aims to satisfy both the clients (thus ensuring the SLA at a fair price), and provider by using the resources available in the system efficiently. Fig 1 shows the proposed module.

A client’s request will be answered by a provider, which uses three layers and can provide different services: application layer (Software as a Service—SaaS), platform layer (Platform as a Service—PaaS) and infrastructure layer (Infrastructure as a Service—IaaS). The application layer handles all the application services that are offered to the clients. The platform layer includes mapping and scheduling policies which are designed to translate the clients’ QoS requirements to infrastructure level parameters and allocating virtual machines to meet their requests. The infrastructure layer carries out the initiation and removal of VMs with specific resource configurations for the client in a transparent way.

First of all, the client and provider must negotiate a SLA, by defining the QoS metrics and the contract details (1). After this procedure, different clients request different types of services from a provider. The **Admission Control** is responsible for analysing the request and decides whether or not it can be met (2). During the system overload, for example, the service provider can decide either to reject the new requests or violate the SLA. The SLA violation should result in penalties for the provider. If the request is accepted, it will be stored in the **Requests Queue** (3), where it will receive a priority that will define the execution sequence (a service differentiation can be applied, for example, with different kinds of clients).

The ReMM will provide the virtual resources based on the information defined in the SLA (4), and place them in the physical resources (5). After the resources allocation, the **Scheduler** forwards the requests (6) so that they can be run on resources using scheduling policies (7.a) (7.b). At intervals of time, the **Performance Monitor** collects information about the system performance and about the request execution (8) and sends them to ReMM (9.a). This information is compared with the QoS information available in the contract (9.b) and, if the results are not in accordance with the SLA, the ReMM dynamically adjusts the amount of resources (horizontally or vertically) in an attempt to ensure the SLA (10). Any changes in the system influence the **Business Model** (11).

Other modules can be included in the environment described, such as a module for prediction, analysis and load balancing, or new scheduling policies that are designed, for example, to assist in energy saving. However, these modules will be addressed in future work. Optimization techniques can also be applied to set the adjustment rate of the changes, which correspond to the resources and the amount of these resources that ReMM must change.

**Fig 2:**
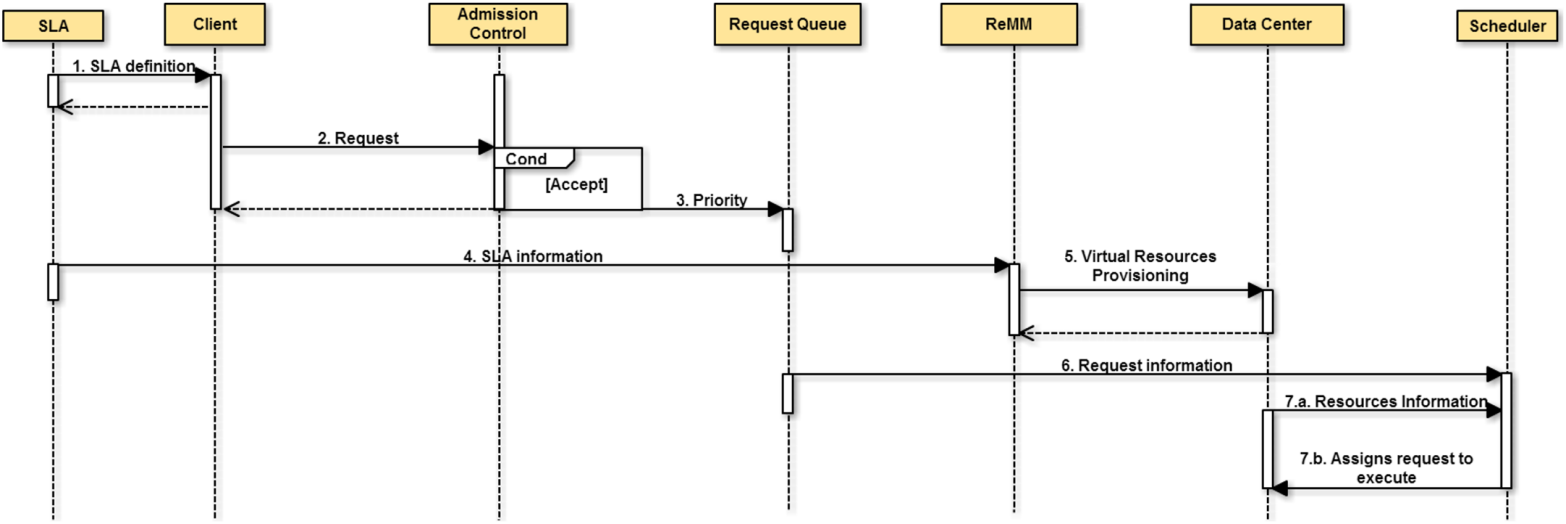
Sequence diagram for processing a request. Services requests will be analysed by the Admission Control after a negotiation between the client and the provider. If a request is accepted, virtual resources are allocated in the physical resources in accordance with the SLA specifications. After this the requests are assigned to run.

**Fig 3:**
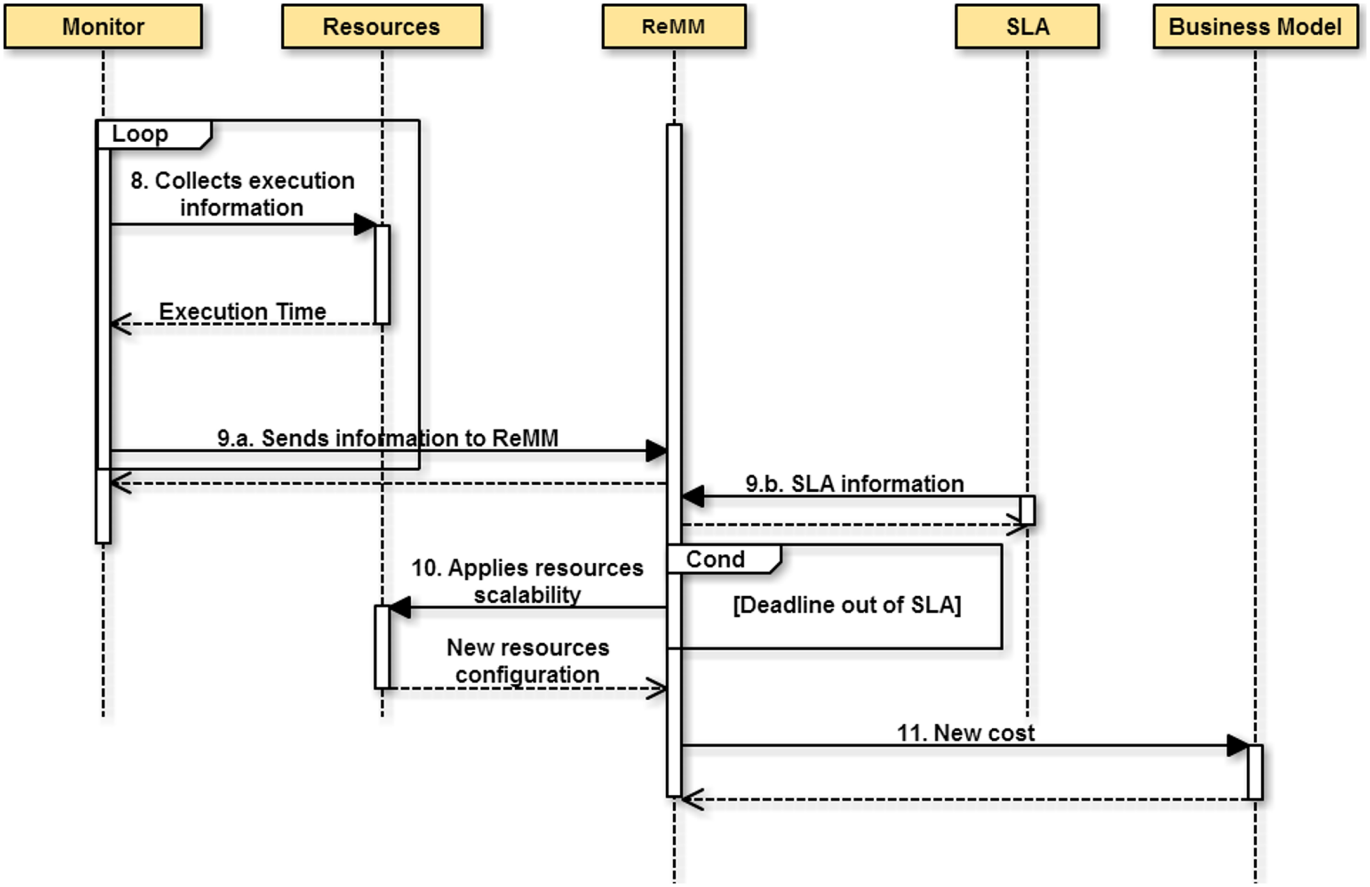
Monitoring and scalability process. At intervals of time, the Performance Monitor collects information about the execution and sends it to ReMM. ReMM analyses the performance information and may or may not apply the resources scalability, which affect the costs.

The sequence diagrams in Figs 2 and 3 show the interactions carried out in response to the client request and the analyses performed to determine whether the resources should be changed or not, and the proportional this has on costs. In the next section, a number of experiments are carried out, which involve an analysis of the behavior of the proposed module.

## Performance Evaluation

In this section, there are two sets of experiments, one using a real and another using a simulated environment. In the first round (Section First Round of Experiments—Analysis for Resource Provisioning), an analysis is conducted to define the computational resources and the amount of these resources that must be allocated to a client in a cloud with different workloads. On the basis of this analysis, we designed a second round of experiments (Section Second Round of Experiments—Simulated Environment with ReMM), in which we show the impact on the system response variables with the changes in the available resources applied by ReMM. The CloudSim 3.0.3 simulator was used to design and implement the cloud environment with ReMM. CloudSim provides the following: modeling and simulated large-scale cloud computing data centers, virtualized server hosts, policies for allocation of hosts to virtual machines and policies for mapping and scheduling applications to virtual machines, i.e., all the necessary entities for resource management [18].

A full factorial experimental planning was used for both sets of experiments. This model is outlined by Jain [19] and is suitable for the analysis of variable responses. In this methodology, the planning and analysis of experiments include both factors and levels, where the factors correspond to environmental characteristics and the levels are the possible environmental variations.

All the experiments were run 10 times, because through 10 repetitions it was noted that the results did not show large variations. Therefore, it was concluded that 10 was a reasonable figure for the number of repetitions.

### First Round of Experiments—Analysis for Resource Provisioning

The aim of the experiments shown in this section is to analyze which computational resources should be provided to improve the performance in a cloud and the proportion in which they should be changed. Two benchmarks were used in the experiments:

1. **Apache**—This System-Bound benchmark uses the number of served requests per second as a response variable [20]. It measures how many requests per second a given system can sustain when carrying out 1000000 requests with 100 requests being carried out concurrently.
2. **Smallpt**—This CPU-Bound benchmark renders an image using a Monte Carlo algorithm and shows the execution time (in seconds) as a response variable [21].

In the experiments with Apache benchmark, five factors with different quantities of levels were taken into account when forming the different scenarios. This information is given in Table 1, where each level was increased by 100% more than the lower level.

**Table 1:** Factors and levels for experiments with Apache benchmark.

Factors	Levels
Disk size	8GB and 16GB
Network type	Megabit and Gigabit
Memory RAM capacity	512MB and 1024MB
VMs number	1, 2 and 4
vCPUs number	1, 2, 4 and 8

After the analysis of the influence of the factors, new experiments were conducted with the Smallpt benchmark, which only involved varying the number of VMs and vCPUs (Table 2) to analyse the behavior of the system with different workloads. The results with both benchmarks show the response variables with a 95% confidence interval.

**Table 2:** Factors and levels for experiments with Smallpt benchmark.

Factors	Levels
Disk size	8GB
Network type	Gigabit
Memory RAM capacity	512MB
VMs number	1, 2, 4 and 8
vCPUs number	1, 2, 4 and 8

In addition, a physical machine was operated that was based on an *Intel Core 2 Quad* processor to host the virtual machines that execute the workload. The environmental configurations are shown in Table 3.

**Table 3:** Environment specification for both benchmarks.

Machine	Physical	Virtual
**Processor**	Core 2 Quad 2.4GHz	Core 2 Quad 2.4GHz
**Cores**	4	Varies
**Memory RAM**	8GB	Varies
**Disk**	160GB	Varies
**Network**	-	Varies
**Operational System**	Ubuntu Server 11.10	Ubuntu Server 10.04
**Hypervisor**	Xen 4.1	-

The way in which the hypervisor combines the physical and virtual resources is an important factor that should be noted in these experiments, since it forms the basis of an efficient resource provisioning. However, this combination may vary depending on the type of hypervisor used. The Xen hypervisor, which implements the Credit Scheduler algorithm, was used for this study [22]. This algorithm considers the total number of vCPUs in the system and divides it between the physical cores. Thus, there are times when, according to the configuration of the experiments, a physical core can be idle or overloaded.

#### Results of the Apache benchmark

**Fig 4:**
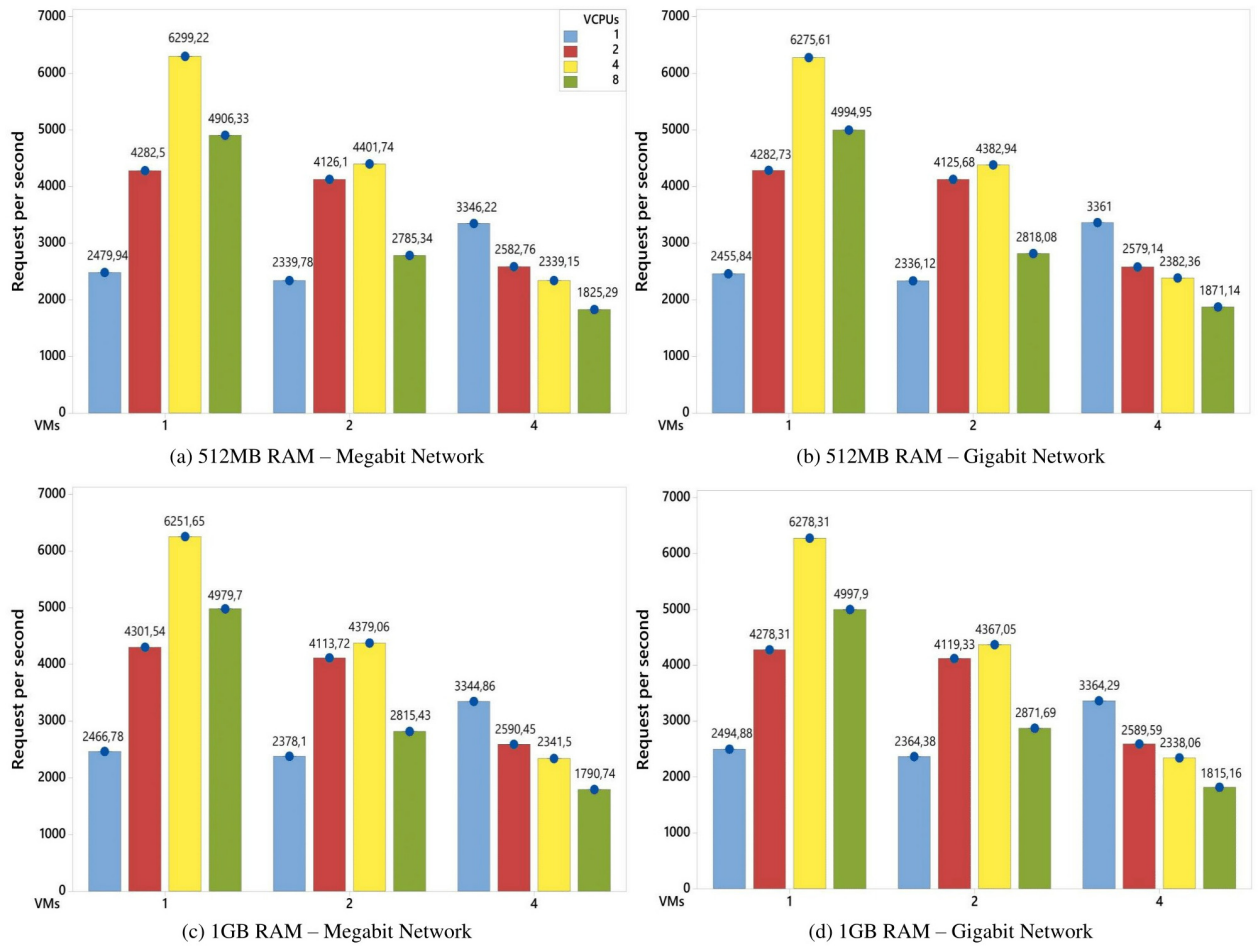
Average number of served requests for each VM in an environment with an 8GB disk. The graphs show the variations of factors when the Jain methodology is employed [19].

Fig 4 shows the average number of served requests (per second) answered by one virtual machine during the experiments execution time. The results show different combinations of levels that include the factors defined in the experimental design (Table 1). However, even with different configurations, the experiments showed almost the same behavior.

According to the graphs, as new VMs were added to the system, the competition for computational resources became greater, and thus reduced the average number of served requests per VM. This behavior was evident when a comparison was made between the experiments with 4 vCPUs and 1, 2 and 4 VMs (yellow columns). In these experiments, there was an increase of 100% in the number of VMs that led to a reduction of, approximately, 30% (1 to 2 VMs) and 46% (2 to 4 VMs) in the number of served requests. No CPU had remained idle since the beginning of the experiments execution.

**Fig 5:**
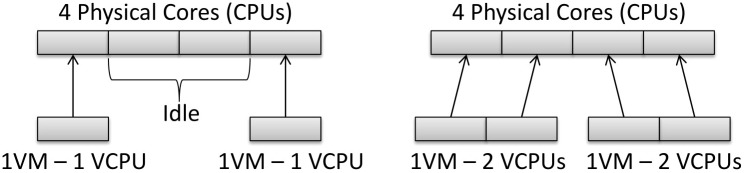
Use of the processor. The way that the Credit Scheduler combines the vCPUs and the CPUs creates situations in which the physical cores can be idle, balanced or overloaded.

On the other hand, the experiments with 1 VM and 1 vCPU had similar results to the experiments with 2 VMs and 1 vCPU. This behavior can be explained by the fact that there were some idle resources during the experiments. This idleness occurred because the number of virtual cores in the VMs was less than the number of available physical cores. The same behavior occurred in the experiments with 1 and 2 VMs and both with 2 vCPUs. However, in the experiment with 1 VM and 2 vCPUs, there was a total of 2 vCPUs that had to be executed in 4 physical cores. In the other case, (2 VMs with 2 vCPUs), there was a total of 4 vCPUs to be executed in 4 physical cores. In this case, each CPU received one vCPU to run and all the vCPUs were executed in parallel. Hence, the results were similar, as the average number of served requests per second per each VM was considered. Fig 5 illustrates this behavior.

Continuing with Fig 4, in the experiments with 4 VMs, the higher the number of vCPUs, the lower the number of served requests per second. For this set of experiments, the number of physical cores was a limiting factor, because the competition for these resources increased as the number of vCPUs increased and, for this reason, there was a reduction in the number of served requests. However, in the experiments with 1 and 2 VMs, the competition for physical resources was lower, and this resulted in a large number of served requests. In the case of 1 VM, the increase of 1 to 2 vCPUs and, later, from 2 to 4 vCPUs in the number of vCPUs increased the response variable by, approximately, 73% and 46%, respectively. In the case of 2 VMs, the same increase in the number of vCPUs resulted in an increase of, approximately, 75% and 6%, respectively.

**Fig 6:**
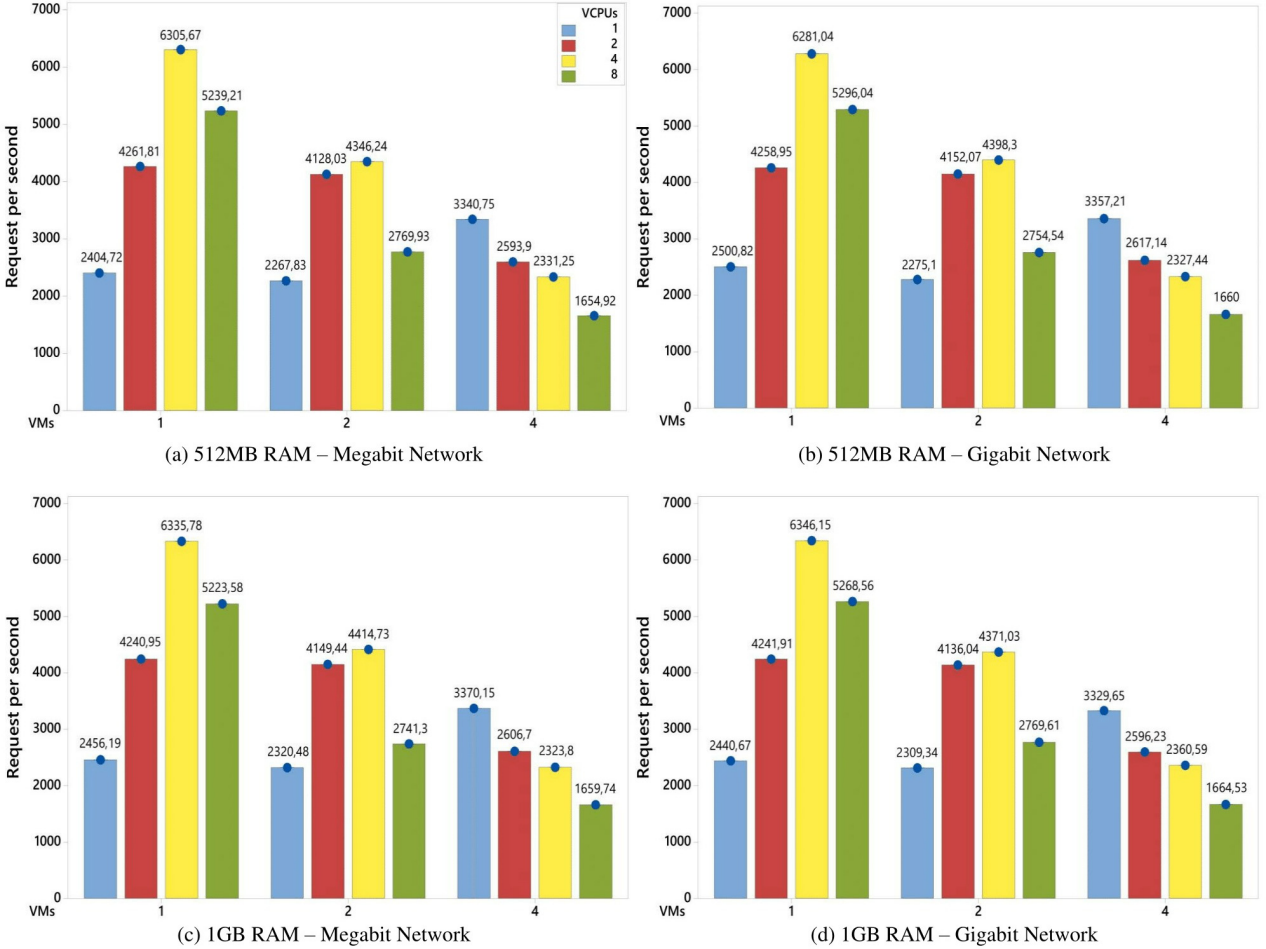
Average number of served requests for each VM in an environment with 16GB disk. The results were similar as shown in Fig 4. This means that, the previous analyses can be applied to these results.

The behavior described in Fig 4 is applied in Fig 6 where the disk size was changed from 8 to 16 GB. In this way, in hte cases of both Figs 4 and 6, the changes in the memory RAM capacity (from 512MB to 1GB), network (from Megabit to Gigabit) and disk size (from 8GB to 16GB) do not lead to significant changes in the results, i.e., the performance of the system remained the same. Thus, when account is taken of the experimental design and the workload used, there were no statistical differences in the results with either a combination or change of these factors. This assertion is discussed in the next section in the analysis of the factors influence.

#### Factors influence

In this paper, a full factorial model was used to measure the influence of each factor and the respective levels on the response variables [19]. In this way, analyses considering the factors Disk size, Network type, Memory (RAM) capacity, VMs number and vCPUs number were performed. Considering the vCPUs number factor has 4 levels, an analysis combining the levels in 2–2 to determine the influence of each factor on the response variable was performed. For the VMs number factor (with 3 levels), the extreme levels were considered, i.e., 1 and 4 VMs.

**Fig 7:**
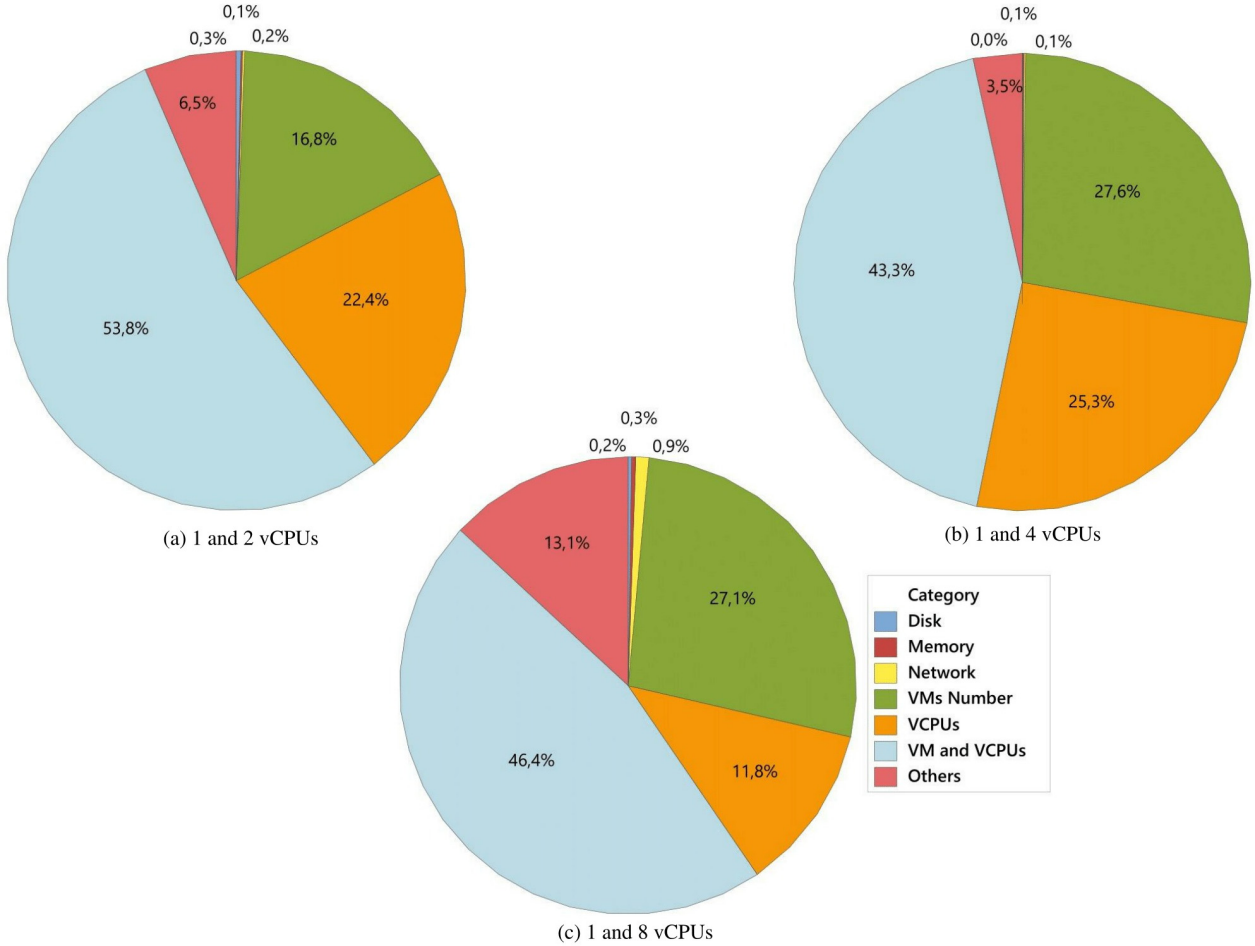
Analysis of the influence of factors. In this analysis the levels in the number of vCPUs factor were combined in 2–2 to define the influence of each factor on the number of served requests per second by taking account of environments with 1 and 4 VMs.

In Fig 7, combination of the number of VMs and vCPUs factors had the greatest influence on the results with 53.8% (Fig 7a), 43.3% (Fig 7b) and 46.4% (Fig 7c). As previously mentioned, as new VMs and vCPUs were added to the system, competition for physical resources became greater, and this had a significant effect on the response variable.

Note that it was only in Fig 7a that the number of vCPUs was the second factor with most influence (22.4%), followed by the number of VMs (16.8%). Through the combinations shown in this graph, it was possible to record situations in which the vCPUs number was lower, equal and greater than the number of VMs. This allowed the percentages of the respective influences to be obtained.

In Fig 7b, although the number of VMs had an influence (27.6%) larger than that of the number of vCPUs (25.3%), the combination of these factors led to a reduction (approximately 19%) in the percentage of influence in Fig 7b when compared to the same combination in Fig 7a. This percentage was added to the percentage of influence of the factors number of VMs and number of vCPUs, which led to an increase of approximately 65% and 13%, respectively. In Fig 7c, the third most influential factor, i.e., the number of vCPUs (11.8%), had a reduction (in terms of the percentage of influence) that was approximately 53% lower than that shown in Fig 7b.

In this way, though the combination shown in Fig 7b, the idleness of processing resources during the experiments was found to be lower than in Fig 7a, while the competition for these resources was also lower than in Fig 7c. Thus given the experimental design and the workload used in the system, the combination of the factors and their levels shown in Fig 7b provided the most efficient use of available resources.

Finally, the percentage of influences of the factors Disk size, Network type and Memory RAM capacity were minimal, as mentioned in the previous section, less than 1% for each one. In view of this, new experiments were performed that were based on these analyses and included a strictly CPU-Bound load based on the Smallpt benchmark, as will be discussed in the next section.

#### Results of the Smallpt benchmark

The previous sections showed that the number of VMs and vCPUs were the factors which had more influence on the response variable when based on a System-Bound workload. These results enabled additional experiments to be performed with a strictly CPU-Bound workload. The purpose of this was to determine if the same behavior is maintained with different workloads. The hardware configuration was the same as that shown in Table 3.

**Fig 8:**
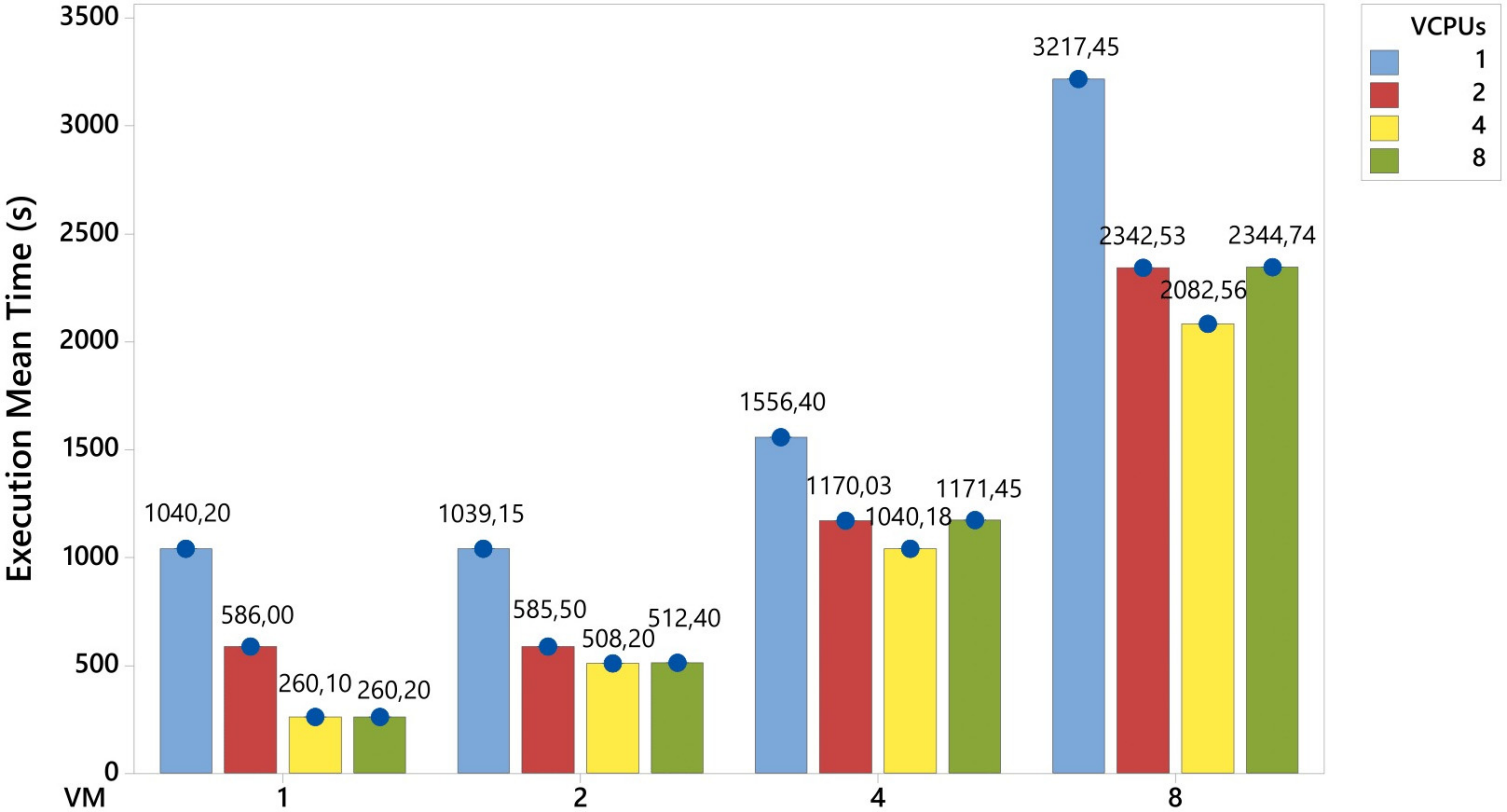
Smallpt analysis. As new vCPUs were added, the competition for physical resources was greater, and this reduced the execution mean time. However this only occurred until the vCPUs number was less than, or equal to, the physical CPU number. After this point, there were increases in the response variable.

In Fig 8, as new VMs were added in the system, the competition for computational resources became greater, and as a result, increased the execution time of the workload. This behavior is shown in the experiments with 4 vCPUs, in which the increase of 100% in the VMs number (from 1 to 2, 2 to 4 and 4 to 8) provided an increase of approximately 95%, 105% and 100% in the response variable, respectively.

An useful way of conducting an analysis involves making a comparison between the columns with the same colors. For instance, although all the experiments represented by the green columns in Fig 8 had a total of 8 vCPUs, the experiments with a smaller VM number had better results. In other words, the experiment with 1 VM and 8 vCPUs obtained a better execution time than the experiment with 2 VMs and 4 vCPUs. This difference was of approximately 49%. When a comparison was made between 1 VM with 8 vCPUs and 4 VMs with 2 vCPUs, the former obtained a lower result than the latter, approximately 78%. In the case of the extremities, i.e., experiments with 1 VM with 8 vCPUs and 8 VMs with 1 vCPU, the difference in the execution time was approximately 92% (when based on a change from 8 to 1 VM). In this way, when there is an environment with the same total number of vCPUs, the combination of 1 VM with 8 vCPUs had a better performance than the combination with 8 VMs and 1 vCPU. The same behavior occurred with environments with 2 VMs with 4 vCPUs and 4 VMs with 2 vCPUs, in which the change from 4 to 2 VMs led to a reduction in the response variable of approximately 57%.

Furthermore, it should be noted that the experiment with 1 VM with 1 vCPU had a similar result to the experiment with 2 VMs with 1 vCPU. This can be explained by the occurrence of idle physical resources in these scenarios while the experiments were being carried out. This behavior was discussed in the analytical experiments described in Figs 4 and 6. The same behavior occurred in the experiment with 2 VMs with 1 vCPU that obtained a similar result to the experiment with 2 VMs with 2 vCPUs. This behavior is illustrated in Fig 5.

Finally, the increase in the vCPU number led to a reduction in the execution time of the system. However, this statement was only valid until the vCPU number reached the physical core number. From this point onwards, the increase in the vCPU number resulted in a greater competition for physical resources, which impaired the system’s performance. When the experiments are analysed with 8 VMs in Fig 8, there is an increase of 100% in the vCPU number, from 1 to 2 and 2 to 4, which led to a reduction in the execution time of, approximately, 27% and 11%, respectively. From this point on, the increase in the vCPU number, from 4 to 8, raised the execution time by, approximately, 13%.

**Fig 9:**
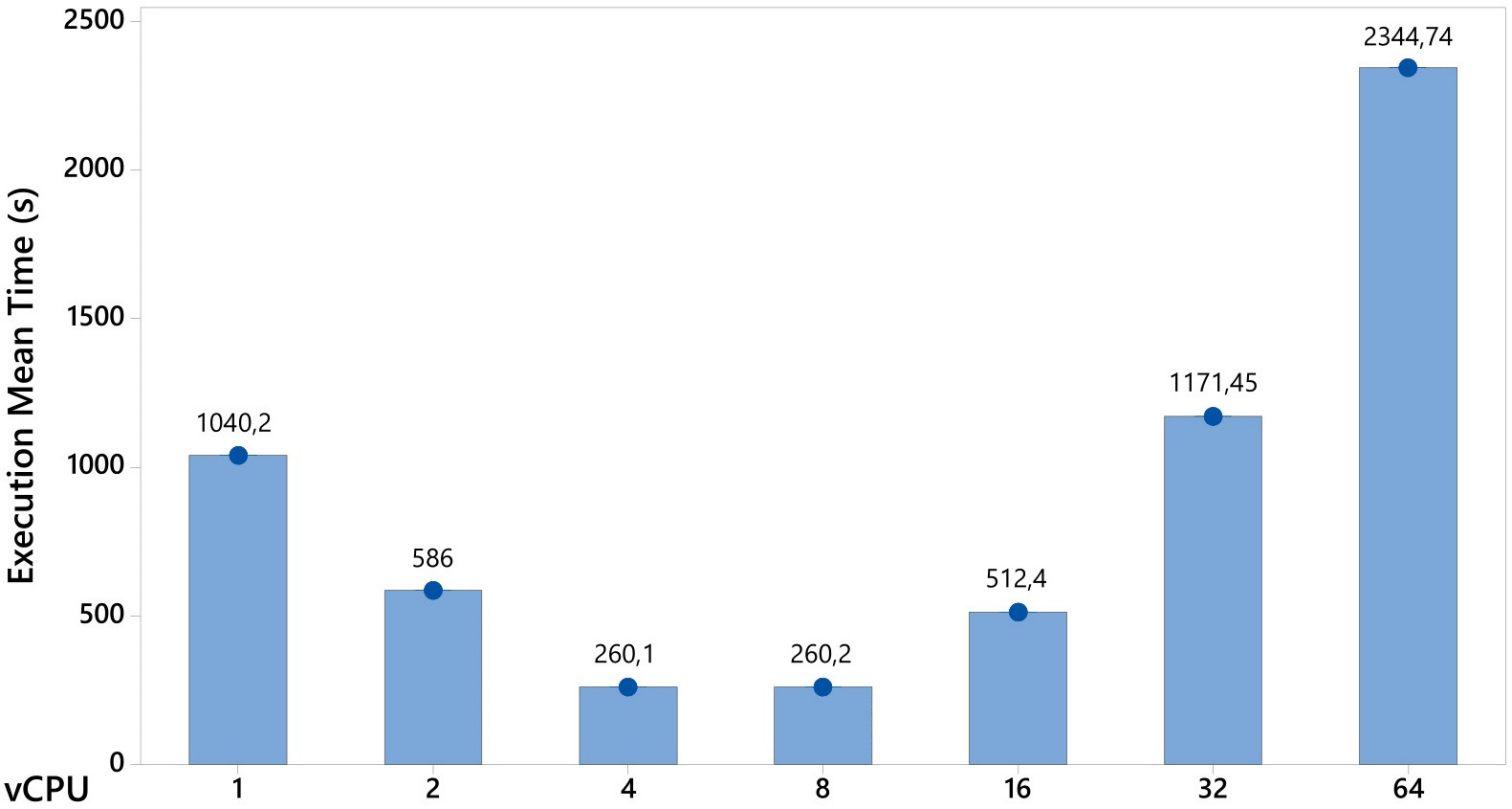
System behaviour with the increase in the number of vCPUs.

Fig 9 shows a comparison that takes account of the increase in the vCPUs number. By conducting this analysis, it is possible to show the described behavior, and that there is an increase in the vCPUs number, from 8 to 16, 16 to 32 and 32 to 64, leading to an increase of approximately 97%, 129% and 100%, respectively, in the response variable.

In these analyses there has been a detailed experimental evaluation to assess the impact of specific resource changes. Different workloads were examined and in nome of them did changes in the memory capacity, disk size or type of network have a significant effect on the response variable. On the other hand, increasing the number of vCPUs rather than the number of VMs proved to be more effective. However, this rate of increase should include the number of available physical cores. In an environment in which the number of vCPUs exceeds the number of physical CPU cores, the environmental performance was impaired, since there was a greater competition for physical resources.

On the basis of the results, only changes in the number of virtual cores were considered in the ReMM implementation. In the vertical scalability, the changes had a percentage of 100%, where 16 vCPUs was the maximum number allowed per VM and 1 vCPU was the minimum. On the other hand, when there was horizontal scalability, the ReMM changed the number of VMs. No account was taken of the physical resources and their limitations this paper. This means the physical resources were regarded as unlimited.

### Second Round of Experiments—Simulated Environment with ReMM

A suitable environment was simulated to evaluate the ReMM. It was assumed that all the physical resources are unlimited, i.e., they are not limiting factors. As a result, all requests can be accepted by the Admission Control. The question of how physical resources can be limited will be examined in future work, and where it is possible to assess the rate of accepted and rejected requests with ReMM, and thus vary the workload demand. Table 4 shows the experimental design.

**Table 4:** Experimental design.

Factors	Levels
VM Instance	*m3.medium*, *m3.large*, *m3.xlarge*, *m3.2xlarge*
Application	Low, Medium, Heavy
Environment	Common, ReMM Horizontal, ReMM Vertical

A client requests an image rendering execution that might be Low, Medium or Heavy, through the Monte Carlo algorithm. This type of service request was modeled on the Smallpt benchmark. Requests can be performed in environments with or without ReMM (Common environment), which are configured with four different types of instances, *m3.medium*, *m3.large*, *m3.xlarge* or *m3.2xlarge*, modeled on Amazon M3 instance types [23]. Each client performs 10 service requests in each experiment and has dedicated resources, i.e., the computing resources allocated to a client are not shared with other clients. Thus, each request is mapped to the corresponding client’s VM. The initial configuration of the VM varies in accordance with the experimental design.

#### Response variables

Two types of response variables will be employed in our module analysis: the execution mean time and the cost. The Execution Mean Time (EMT) quantifies the performance of requests by measuring the average time spent running the requests (in seconds). Eq (1) represents the EMT. The cost (in dollars) is defined according to the instance and this changes as the ReMM modifies the resource configurations on-the-fly.

$$EMT=\sum_{i}^{10}\frac{TaskExecutionTime_{i}}{NumberofTasks}$$ (1)

**Fig 10:**
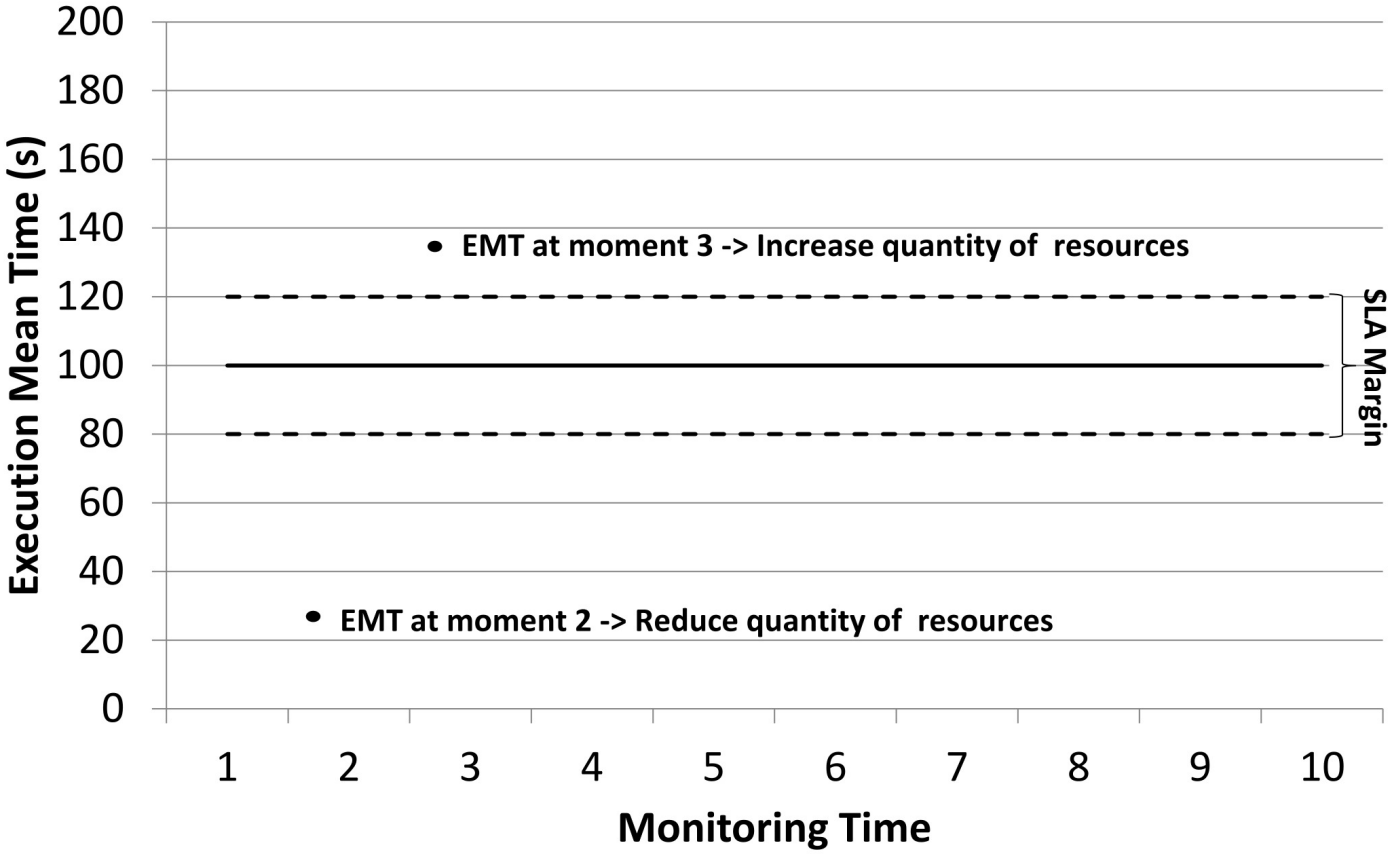
SLA Margin. The EMT is collected and analysed at intervals of time. The resources might be changed, depending on the results of the analysis.

A SLA Margin is defined in the contract signing, which represents the variance-based performance measurement. Its behavior is shown in Fig 10. In the experiments, the client requests the image rendering (which might be Low, Medium or Heavy) in approximately 100 seconds, with a SLA Margin of 20%, i.e., the request execution time can vary from 80 and 120 seconds. The ReMM analyses the EMT in periods of time and, if the result is not in accordance with the SLA Margin, it changes the amount of resources allocated for that client.

In Fig 10 the EMT at moment 2 is smaller than the contracted performance. This means that the ReMM must reduce the VM resources during the execution time so that the next request will be able to run with a different resource configuration. This change is necessary as the current configuration, in this example, is using more resources than needed. Thus, the client will be expected to pay more and his/her request will not be completed in the required time. At moment 3, there is a situation in which the resources are not enough to complete the request in the appropriate time, because the EMT is outside of the SLA Margin, and hence the ReMM has to obtain an increase in resources in an attempt to reach the contract.

As a result, the proposed module seeks to ensure the requested execution time is met by respecting the SLA Margin and changing the resources configuration, if necessary, with a corresponding change in price. The ReMM can use both vertical and horizontal scalability to achieve this.

#### Results and discussion

**Fig 11:**
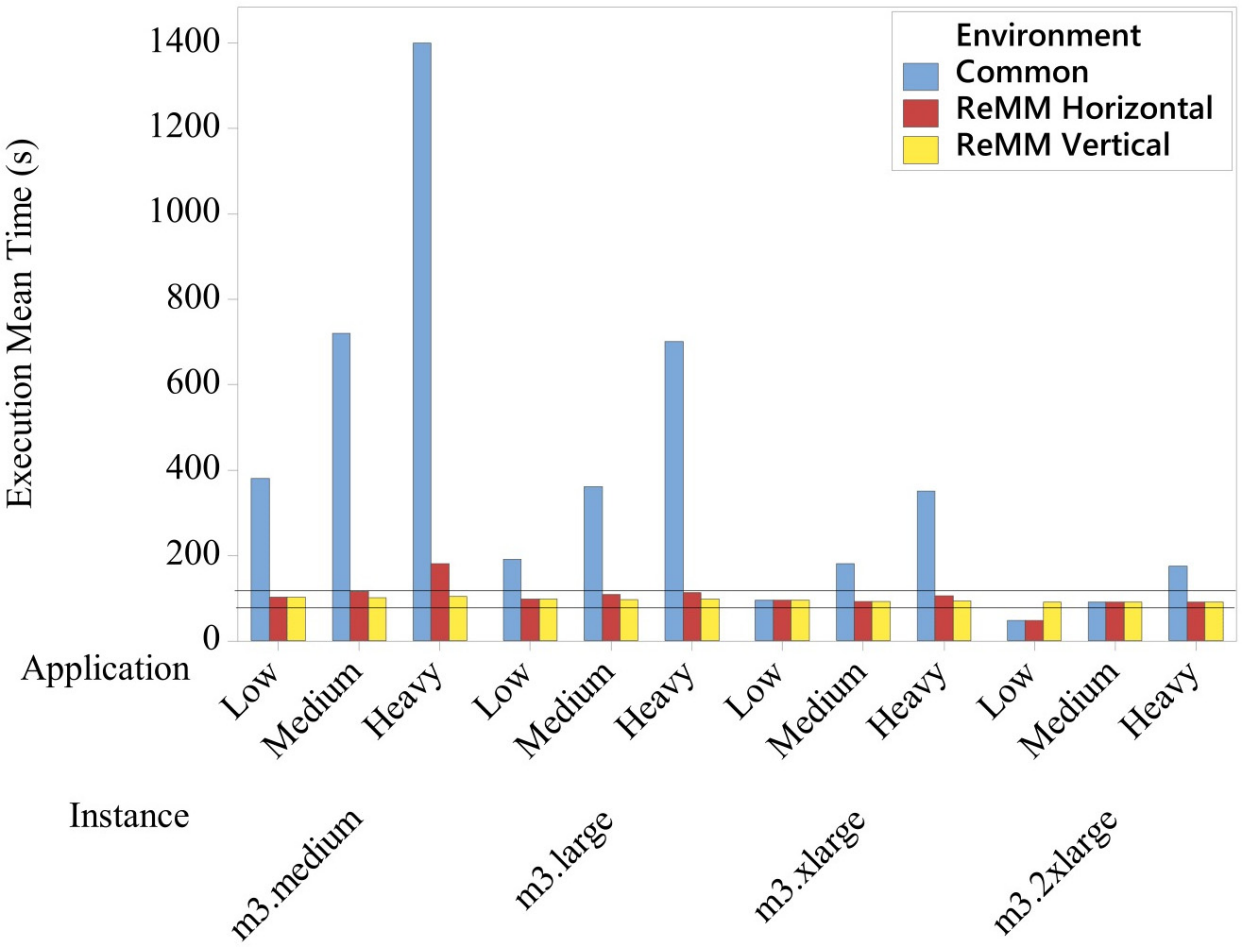
Results of the Execution Mean Time. Different applications were executed by different instances in environments with and without ReMM.

Fig 11 shows the results obtained from the experimental design described in the previous section. The vertical and horizontal scalability were applied in these experiments and compared with a Common environment without ReMM, by measuring the execution mean time in seconds, and estimating the cost in dollars. The results showed that the execution mean time increased as the application complexity increased. On the other hand, as the VM instance became more powerful, there were reductions in the execution mean times.

The amount of resources available in the *m3.medium* instances in the Common environment was insufficient for the execution of applications within the SLA (remembering that a client required an execution time of around 100 seconds with a SLA Margin of 20%). Hence, changes in the number of vCPUs and VMs were necessary.

Apart from the experiments with *m3.medium* instances running Heavy applications and with *m3.2xlarge* instances running Low applications, both with horizontal scalability, the ReMM fulfilled all the signed contracts (Table 5 shows all the values). In the first case, the initial amount of resources available on *m3.medium* instances (column Resources—Common vCPUs) was found to be too small for the execution of Heavy applications, and resulted in an execution mean time of 180.6s. Additions of new *m3.medium* instances were necessary, and this led to higher cost and reduced the execution mean time. However, the rate of change used in the horizontal scalability was not enough to ensure the SLA was complied with before the end of the execution. In this way, 9 changes (column Changes—Horiz.) in the number of instances were carried out. In the second case, the amount of resources available in the *m3.2xlarge* instance for the execution of Low applications was considered to be excessive (8 vCPUs). In view of this, the ReMM attempted to reduce the number of *m3.2xlarge* instances but was unable to, because the minimum number of instances had been achieved (column Resources—Horiz. VMs).

**Table 5:** Results of the experiments.

Design		EMT			Resources					Changes		Cost per Hour ($)		
Instances	Application	Common	Horiz.	Vert.	Common vCPUs	Horiz. vCPUs	Vert. vCPUs	Horiz. VMs	Vert. VMs	Horiz.	Vert.	Common	Horiz.	Vert.
*m3.medium*	Low	380	102.7	101.3	1	4	4	4	1	3	2	0.1082	0.4328	0.1352
	Medium	720	115.2	100.7	1	8	8	8	1	7	3	0.1082	0.8656	0.1712
	Heavy	1400	180.6	103.3	1	10	16	10	2	9	4	0.1082	1.0820	0.3424
*m3.large*	Low	190	97.4	97.4	2	4	4	2	1	1	1	0.2166	0.4332	0.2346
	Medium	360	107.5	96	2	8	8	4	1	3	2	0.2166	0.8664	0.2706
	Heavy	700	112.0	97.9	2	16	16	8	2	7	3	0.2166	1.7328	0.5412
*m3.xlarge*	Low	95	95	95	4	4	4	1	1	0	0	0.4387	0.4387	0.4387
	Medium	180	92.3	92.3	4	8	8	2	1	1	1	0.4387	0.8774	0.4747
	Heavy	350	104.5	93.3	4	16	16	4	2	3	2	0.4387	1.7548	0.9494
*m3.2xlarge*	Low	47.5	47.5	90.5	8	8	4	1	1	0	1	0.8775	0.8775	0.8415
	Medium	90	90	90	8	8	8	1	1	0	0	0.8775	0.8775	0.8775
	Heavy	175	89.7	89.7	8	16	16	2	2	1	1	0.8775	1.7550	1.7550

**Fig 12:**
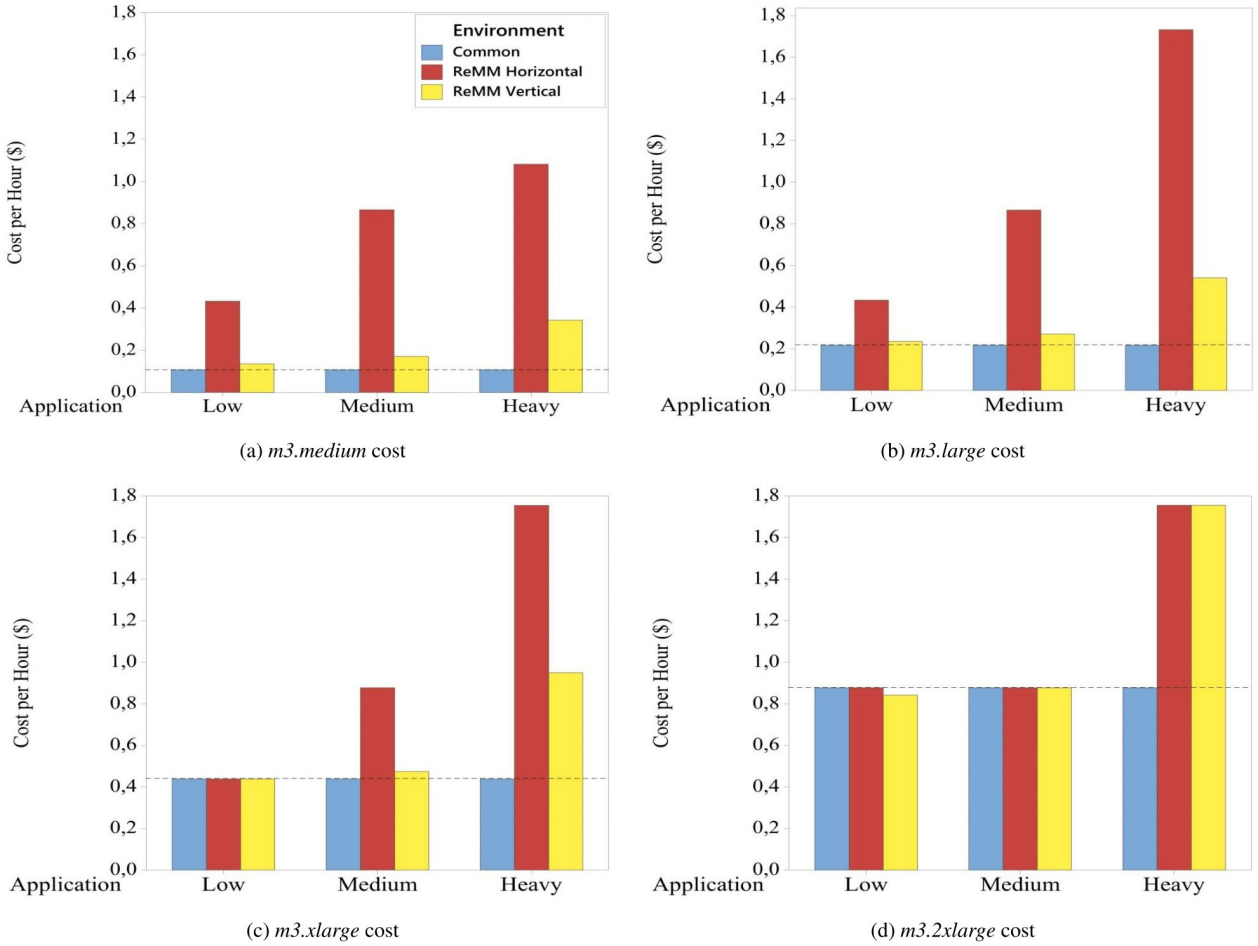
Cost per hour. In vertical scalability the costs were changed as the vCPUs number was changed. On the other hand, in horizontal scalability the costs were changed proportionally to the number of instances.

In view of the vertical scalability, the changes in the vCPUs led to an increase of approximately 25%, 58% and 216% in the cost (Fig 12a) during the execution of Low, Medium and Heavy applications, respectively, by *m3.medium* instances. However, for the same applications, the changes in the number of vCPUs reduced the execution mean times by approximately 73%, 86% and 93%, respectively, which made these changes essential for compliance with the SLA. With regard to horizontal scalability, the changes in the number of *m3.medium* instances led to reductions in the execution mean times of Low, Medium and Heavy applications, approximately 73%, 84% and 87%, respectively. However, these changes influenced in the costs and caused rises of, approximately, 300%, 700% and 900%, respectively.

The changes applied by the vertical and horizontal scalability in *m3.large* instances yielded similar results when both were compared with the Common results. The vertical scalability applied an increase in the number of vCPUs of 100% for running Low, 300% for Medium and 700% for Heavy applications (columns Resources—Common and Vert. vCPUs). According to Table 5 and Fig 11, this increase in the vCPUs led to reductions in the execution mean times of approximately 49% (Low), 73% (Medium) and 86% (Heavy). On the other hand, the use of horizontal scalability resulted in rises in costs of approximately 100%, 300% and 700%, respectively. The reason for this was that the number of changes in this environment was greater than the other ones (column Changes); as well as this horizontal scalability changes the number of VMs, whereas vertical scalability only changes the number of vCPUs (Fig 12b).

In *m3.xlarge* instances, the amount of available resources for running Low applications was considered to be sufficient in all the environments. Accordingly, the ReMM did not change the *m3.xlarge* instances with Low applications. This means that, the execution mean times and the costs (Fig 12c) were the same. On the other hand, the increase in the application required an increase in the amount of resources in the other experiments, 100% for Medium and 300% for Heavy applications, in both the vertical and horizontal environments. In the case of vertical environments, the increase in vCPUs resulted in a rise in costs of approximately 8% (Medium) and 116% (Heavy), and reductions in the execution mean times of 49% for Medium and 73% for Heavy applications. In the case of horizontal environments, the rise in costs was approximately 100% (Medium) and 300% (Heavy), while the reduction of the execution mean times was 49% for Medium and 70% for Heavy applications.

With regard to *m3.2xlarge* instances, the Low application EMT using a Common environment was lower than in an environment in which the vertical scalability was used (47.5 and 90.5 seconds, respectively). Although this execution mean time in a Common environment was approximately 47% lower, the SLA was not complied with. For this reason, the ReMM reduced the number of vCPUs by 50% (from 8 to 4), plus the cost by 4% (Fig 12d), to ensure compliance with the SLA (Fig 11). The best alternative for running a Low application in approximately 100 seconds is the allocation of a *m3.xlarge* instance rather than a *m3.2xlarge*. In a *m3.xlarge* instance, changes in the amount of resources are not necessary and there is a reduction in costs of approximately 50%, from $ 0.8775 (*m3.2xlarge*) to $ 0.4387 (*m3.xlarge*).

In Figs 11 and 12d, the amount of available resources in *m3.2xlarge* instances for the execution of Medium applications was sufficient in all the environments. This meant that, no changes were made in the number of vCPUs and instances, resulting in the same values for the execution mean times and costs. On the other hand, the application change, from Medium to Heavy, required an increase of 100% in the resources in the vertical and horizontal environments, resulting in a reduction of approximately 49% in the execution mean time and an increase of 100% in the cost.

**Fig 13:**
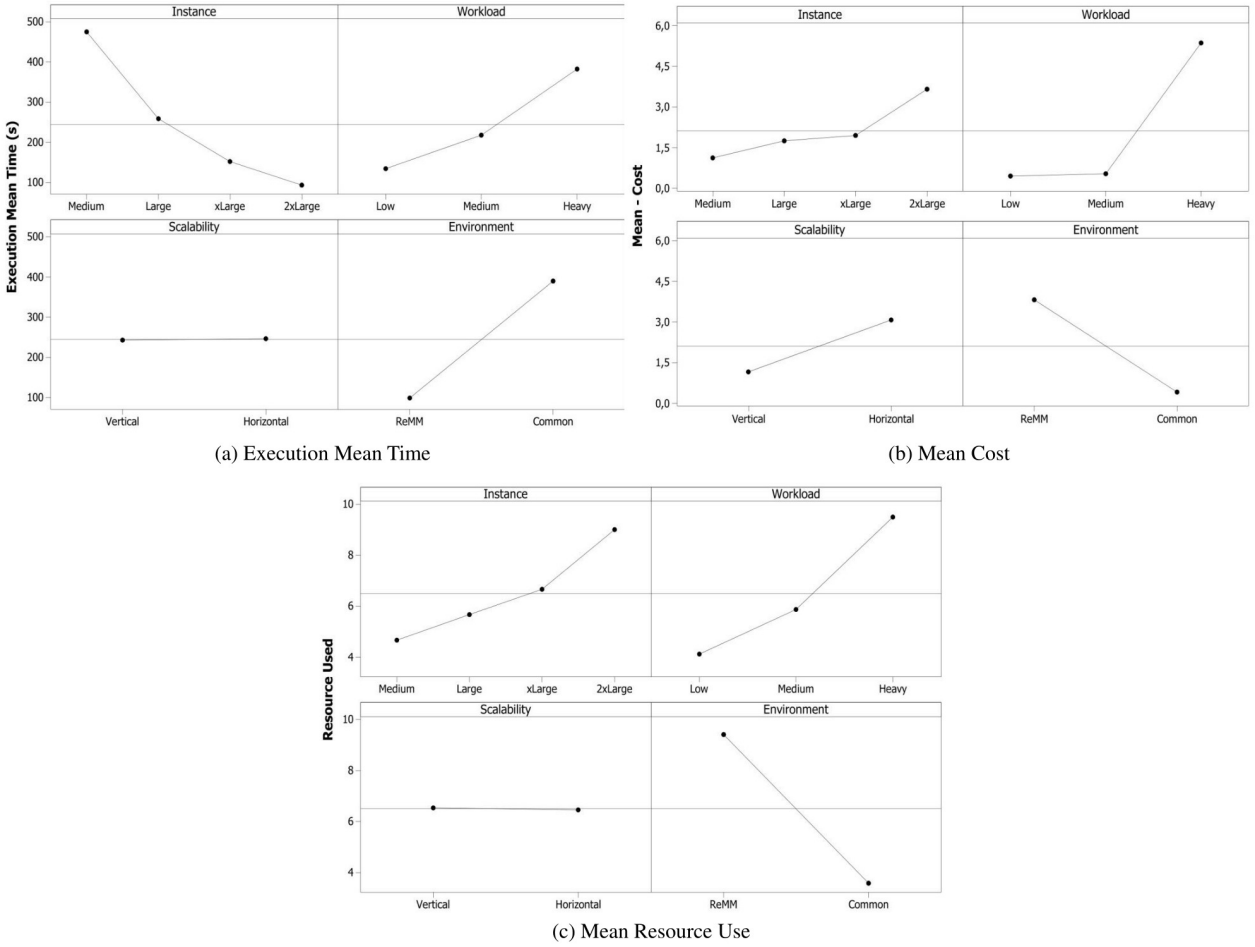
Factors analysis. As the power of the instance increased, the EMT was reduced. This behavior has a direct bearing on the cost and the amount of resources that are used.

In Fig 13 it is possible to analyse the behavior of the response variables while taking account of environmental factors. In the ReMM, the change in the cost when vertical scalability is used is proportional to the change in the number of vCPUs, while in the case of horizontal scalability, the scalability type that most providers use, the change in the cost is proportional to the number of instances. Although the numbers of vCPUs in both vertical and horizontal scalability are the same, apart from the exceptions discussed in this section, the number of allocated instances has a strong influence on costs. For this reason, the cost when horizontal scalability was used, was higher in all the cases when the amount of resources was changed.

## Conclusion and Future Work

This paper has outlined a performance evaluation of resource management in a cloud environment. We proposed ReMM, which is a dynamic and self-managed module that aims to ensure the QoS that is contracted by a client and to use the available resources efficiently. For reason, it changes the available resources on-the-fly, using both horizontal and vertical scalability with an appropriate adjustment to the price, when necessary.

Some experiments were carried out with the aim of measuring the computational resources and the amount of these resources that have to be allocated to a specific client to maximize the resource utilisation and make improvements in performance.

In the results, changes in the memory capacity, disk size and network type did not have a significant impact on the response variable. On the other hand, it was found to be more efficient to increase the number of vCPUs rather than the number of VMs. However, as shown in Fig 9, this increased rate should take account of the number of available physical cores. In an environment in which the number of vCPUs exceeded the physical CPU cores, the environmental performance was impaired, since the competition for physical resources was greater.

After these analyses had been conducted, new experiments were carried out with the aim of analysing performance behavior in a system using ReMM. The results showed that ReMM effectively changed the available resources during the execution time, ensured compliance with the SLA and the efficient use of resources at a fair price.

The next stages will entail analysing and formulating policies and methodologies for the admission control (where different priorities of clients can be considered); workload prediction, load balancing and optimization metrics. A testbed will be examined in the next analysis with different workloads.
